# Knowledge-Based Query Construction Using the CDSS Knowledge Base for Efficient Evidence Retrieval

**DOI:** 10.3390/s150921294

**Published:** 2015-08-28

**Authors:** Muhammad Afzal, Maqbool Hussain, Taqdir Ali, Jamil Hussain, Wajahat Ali Khan, Sungyoung Lee, Byeong Ho Kang

**Affiliations:** 1 Department of Computer Engineering, Kyung Hee University, Seocheon-dong, Giheung-gu Yongin-si, Gyeonggi-do 446-701, Korea; E-Mails: muhammad.afzal@oslab.khu.ac.kr (M.A.); maqbool.hussain@oslab.khu.ac.kr (M.H.); taqdir.ali@oslab.khu.ac.kr (T.A.); jamil@oslab.khu.ac.kr (J.H.); wajahat.alikhan@oslab.khu.ac.kr (W.A.K.); 2 Department of Computing and Information Systems, University of Tasmania, Hobart 7001, Australia; E-Mail: byeong.kang@utas.adu.au

**Keywords:** automated query construction, knowledge-based queries, CDSS, Arden Syntax, medical logic modules

## Abstract

Finding appropriate evidence to support clinical practices is always challenging, and the construction of a query to retrieve such evidence is a fundamental step. Typically, evidence is found using manual or semi-automatic methods, which are time-consuming and sometimes make it difficult to construct knowledge-based complex queries. To overcome the difficulty in constructing knowledge-based complex queries, we utilized the knowledge base (KB) of the clinical decision support system (CDSS), which has the potential to provide sufficient contextual information. To automatically construct knowledge-based complex queries, we designed methods to parse rule structure in KB of CDSS in order to determine an executable path and extract the terms by parsing the control structures and logic connectives used in the logic. The automatically constructed knowledge-based complex queries were executed on the PubMed search service to evaluate the results on the reduction of retrieved citations with high relevance. The average number of citations was reduced from 56,249 citations to 330 citations with the knowledge-based query construction approach, and relevance increased from 1 term to 6 terms on average. The ability to automatically retrieve relevant evidence maximizes efficiency for clinicians in terms of time, based on feedback collected from clinicians. This approach is generally useful in evidence-based medicine, especially in ambient assisted living environments where automation is highly important.

## 1. Background and Introduction

Evidence-based practice [1,2] has a long history, but evidence indicates that many opportunities to use research to inform health decision-making are currently being missed [3,4,5]. For any evidence-based system to efficiently work in a domain, the context of that domain plays a critical role. Context provides the features for query generation in order to approach relevant information. A clinical decision support system (CDSS) can be considered one of the potential sources to be employed for automatic query construction to retrieve research evidence from online resources. CDSSs are widely used around the world [6,7], and “Meaningful Use” regulations for electronic health records (EHR) [8] considered CDSS an essential feature of EHR. Every CDSS has at least two core components: the knowledge base (KB) and inference engine, where the KB is built using multiple approaches ranging from data-driven to expert-driven approaches. The strength of the KB is not determined from the generating approach *per se*, but rather from the effectiveness of how it is developed [9]. Clinical information needs are considerable, and diagnostic knowledge in KB of CDSS cannot provide all necessary descriptive details, which need to be supported with research evidence found in external resources. External resources are abundant, with more than 750,000 biomedical articles published in MEDLINE in 2014 [10].

To access the MEDLINE databases, different search engines have been developed, such as PubMed [11] and HubMed [12]. These engines provide instant access to biomedical literature; however, the retrieval set includes a massive amount of citations [13]. Also, these engines require queries to be manually written. In manual query creation, users with varied experience create queries and retrieve the results. PubMed supports keyword-based search using Boolean operators [14]; however, information needs cannot always be expressed in simple keywords, and sometimes, a question format is superior. The query strength is subject to the expertise of the creator. Queries by expert clinicians differ from those designed by inexperienced clinicians. Moreover, the structure of a query varies from one user to the next, which affects the results.

In the clinical domain, some advances in information mining include Infobuttons, first defined by Cimino as information retrieval tools that automatically generate queries to e-resources using contextual information [15] and patient data from electronic medical records (EMR) [16,17,18,19,20]. The main focus of the Infobutton approach is to establish context-specific links to health information e-resources. Infobuttons are based on topic-based linkages to the e-resource from within the context of an EMR. The query topics are pre-specified, and the user only needs to click on a specific Infobutton placed next to the topic. The query is then generated from the terms associated with the chosen topic, and potential external e-resources are returned, such as UpToDate [21], MedlinePlus [22], PubMed, and others. SmartQuery [23] extracts patient data from electronic patient records and builds a query to provide context-sensitive links to relevant medical knowledge sources. The issue with using electronic patient records for query construction is the lack of relationships among chosen terms. Recently, CDAPubMed [13] has proposed an extension to a Mozilla Firefox browser that semi-automatically generates queries from EHR and filters with relevant terms including MeSH terms. This tool utilizes the HL7 clinical document architecture (CDA) to identify relevant terms from different sections and generate a query accordingly. Both InfoButtons and CDAPubMed use electronic patient records to generate queries that are then further refined by the users.

We address this issue by utilizing the CDSS KB instead of the electronic patient record in order to minimize user involvement during query construction. The CDSS KB imparts knowledge about patients in the form of rules including conditions (If part) and results (Then part), which allow the creation of knowledge-based queries covering both patient context and user objectives. Unlike conventional CDSS KBs, we tested the methods on a standard knowledge base of Smart CDSS encoded in a medical logic module (MLM) with Arden Syntax language [24]. Arden Syntax provides the opportunity to build a KB consisting of a set of units known as MLMs, each of which contains sufficient logic for at least one medical decision [25]. Arden Syntax is a recognized standard for representing clinical knowledge that is both understandable by humans and interpretable by systems and is used for alerts and recommendations in clinical decision support. The Arden Syntax baseline was established in 1992 by the American Society for Testing and Materials and was adapted later in 1999 by HL7. It provides a standard base format that resembles natural language which make it easier to understand for non-experts [24].

Previously, we worked on evidence support for Smart CDSS [26] by designing KnowledgeButton [27], a model for evidence monitoring and adaptation from online knowledge resources in the context of CDSS. It is now integrated with Smart CDSS in the domain of head and neck cancer and includes four key components of query management, communication management, evidence management, and rule management. This paper focuses on the query management part of KnowledgeButton in order to generate knowledge-based queries from the knowledge slot of MLMs.

This paper is organized as follows. Section 2 presents the role of evidence support in ambient assisted home care environment. Section 3 describes the methods for query construction. Section 4 presents the evidence-supported treatment recommendation scenario with experimental results and evaluation. Section 5 presents a case study of evidence-supported medication recommendation in ambient assisted home care environment. Section 6 discusses the findings and limitation of the work, and Section 7 concludes the work.

## 2. Evidence Support in Ambient Assisted Living

**Figure 1:**
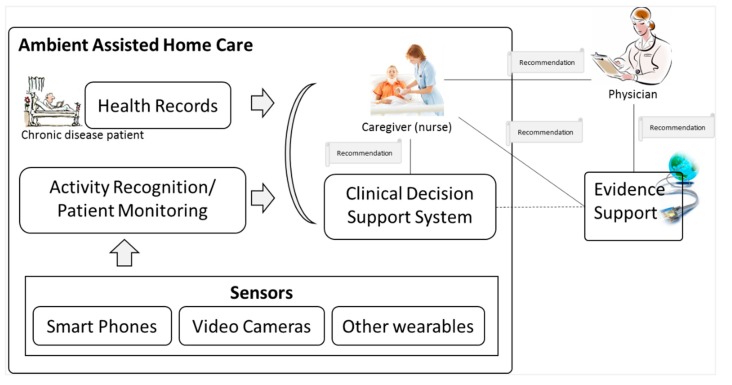
Evidence supported CDSS recommendation service for chronic disease patients in ambient assisted home care environment.

Ambient intelligence and ubiquitous computing technologies are driving the swift evolution of concepts, principles, architectures and techniques in decision support environments [28]. Decisions are derived from situations, and situations require confidence in implementing decisions. The prime source for confidence in these critical situations is the availability of evidence at the point of decision. Evidence-support services help with ambient intelligence in smart environments for employing quality decision-making to improve the life care of the users. This includes resolving the challenges in the ambient assisted ubiquitous environments, which become a real challenge for the implementers due to consideration of multiple factors induced by the multi-model sensors with context-aware environment. As described in Figure 1, the caregiver (nurse) is taking care of chronic disease patients monitored through sensors, and the recommendations are generated automatically by the CDSS. The recommendations are reviewed by the nurse and sent to the physicians for approval prior to action. The recommendations are supported with evidence retrieved from online credible resources triggered at two levels:

- Caregiver fails to receive a response from physicians in a specific time period; he/she consults relevant evidence to make sure that the recommendations are appropriate in the current situation.
- Physician monitors the evidence support related to a particular recommendation for confidence building and to consolidate the decision process.

Figure 1 shows the scenario for evidence support by implementing automated construction of knowledge-based complex queries using the patient information in knowledge-based rules. By knowledge-based complex queries we mean queries that consist of one or more concepts extracted from the knowledge rules of CDSS. We collected information of chronic disease patient that includes head and neck cancer and dementia. Two application scenarios are described in this paper based on these two types of chronic diseases;

(1) Evidence-supported treatment recommendation service for chronic disease (head neck cancer patients)
(2) Evidence-supported medication recommendation service for chronic disease (dementia patients)

The empirical results of the proposed idea are based on the experiments performed using clinical rules and a gold standard corpus of annotated documents for the scenario of head and neck cancer. In the same way, the second scenario is described with a case study presenting a detailed workflow for integrating evidence support for managing medication of patient in a home health environment.

## 3. HL7 Arden Syntax and Medical Logic Module

HL7 Arden Syntax is an open standard of medical knowledge representation where the medical knowledge is represented in the form of modular logic unit called a Medical Logic Module (MLMs) which is sharable across the organization [29]. HL7 Arden Syntax specifies the knowledge in the form of independent rules, formulas, or protocols in an amenable set of MLMs. An MLM is group of slots organized into three main categories and one optional category: maintenance, library, knowledge, and resources (optional).

**Figure 2:**
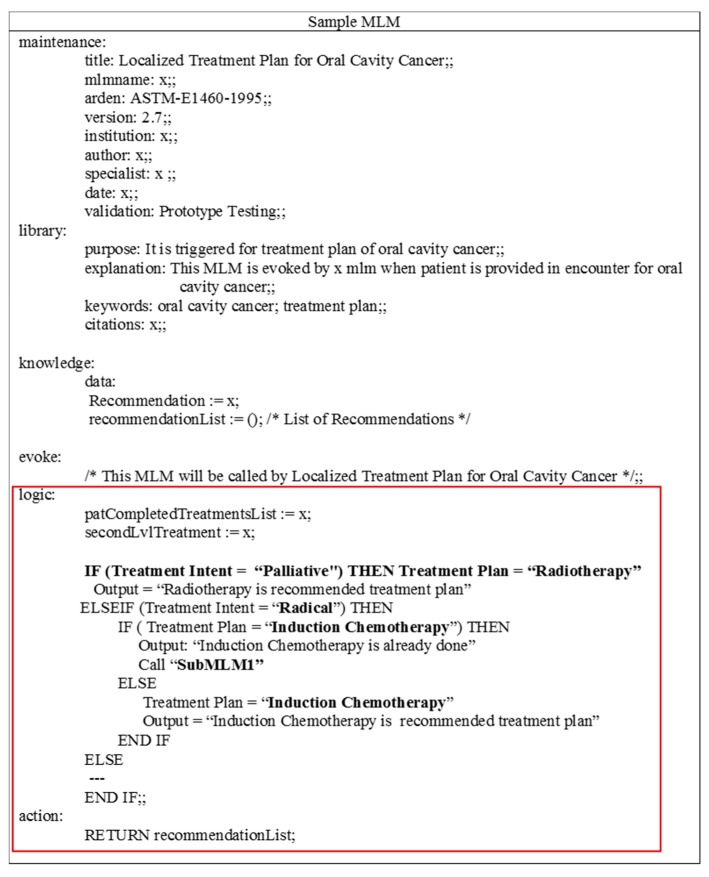
Sample MLM for oral cavity cancer with highlighted “logic” slot in the knowledge category.

The “maintenance” category includes sub-slots used for maintenance and change control such as “MLM title”, “author”, “Arden Syntax version” and other related information. The “library” contains sub-slots that help in searching through the knowledge base of MLMs such as “purpose”, “keywords”, and “citations”. The “knowledge” specifies the intention of what the MLM does. Its sub-slots include a “data” slot, “evoke” slot, “logic” slot, and “action” slot. The “resource” is an optional category that contains a set of slots to support localize messages of recommendations in different languages. Detail specifications with the current version 2.10 of HL7 Arden Syntax are available in the HL7 Arden Syntax working group repository [30].

Among MLM categories, the “knowledge” category is more relevant to this work. In the knowledge category, the more important slot is “logic” that provides sufficient knowledge to construct knowledge-based queries for evidentiary support. The general approach in the logic slot is to use the operators and expressions to manipulate the patient data obtained in the data slot in order to test for some condition in the patient. It mainly consists of control structures such as conditional structure “if-then, if-else-then, *etc.*”, selective structure “switch”, loop structure “for, while, *etc.*”, assignment statements, call statements and others. The information in the logic slot is connected through different operators such as comparison operators to compare a concept with its values. A sample MLM is partially depicted in Figure 2, where the logic slot of knowledge category is highlighted. In this sample MLM, some of the control structures such as “if-then”, “elseIf-then”, “if-else-then” are used. Also, there is a comparison operator “=” and call statement “call: submlm1”. In the Methods section, we described the process of parsing the control structures and operators.

## 4. Methods

**Figure 3:**
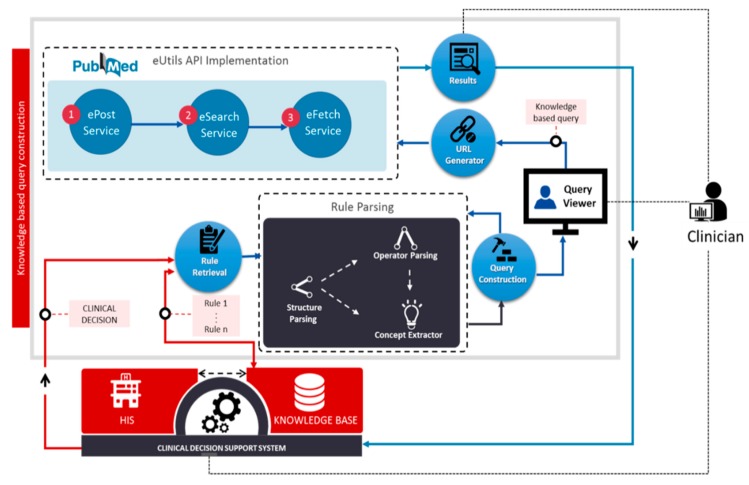
Knowledge-based query construction using CDSS rules.

The knowledge-based query construction proposed here and illustrated in Figure 3 uses CDSS knowledge rules. The process is activated when CDSS is triggered to generate some output, which is used as the input to the query construction process. The rule retrieval function retrieves all the rules (MLMs) that were used in the decision. The CDSS output message is used as the input to determine the exact location in the MLM logic slot at which parsing must begin. Parsing includes two functions, control structure parsing and operator parsing. The logic slot of an MLM has multiple alternative paths because it has different conditional structures as explained in Section 3; however, for a given decision, only one logical path should be utilized.

### 4.1. Structure Parsing

In this step, the control structures used in the identified section are parsed. According to Arden Syntax, a number of variations of If-Then statements are used in the logic slot, such as Simple If-Then statement, If-Then-Else statement, If-Then-Elseif statement, Switch statement, Call statement, and others. Each of these statements is parsed according to the methods described below.

- Simple If-Then statement: The parsing process divides such statements into two sentences, the If sentence and the Then sentence. The concepts found in the If sentence are recognized as condition concepts, and concepts in the Then part are recognized as decision concepts. (See parsing example in Table 1(A));
- If-Then-Else statement: Such statements are parsed into three sentences. If a condition is satisfied in the If part, then the parsing is like a Simple If-Then statement. However, if a recommendation is found in the Else part, then the associated concepts are considered to be decision concepts, while the condition concepts in the If part are negated. (See parsing example in Table 1(B));
- If-Then-Elseif statement: Unless it is a last Else part, such statements are treated similar to a simple If-Then statement, with Elseif similar to If. The last Else part is handled similar to an If-Then-Else statement by considering Elseif as similar to If. For simplicity and to avoid multiple negations due to more than one ElseIf statement, we scoped the parsing to include immediate Elseif only. (See parsing example in Table 1(C));
- Nested If-Then statements: Sometimes an If statement occurs inside another If statement. In such cases, we consider the inner and outer statements as two conditions. For example, if a Simple If-Then statement occurs in another Simple If-Then statement, it is parsed into three sentences, If, If, and Then. Concepts in both if sentences are included in condition concepts, while concepts of the then sentence are included in decision concepts. (See parsing example in Table 1(D));
- Switch statement: The only case involving recommendation is the required segment where the concept value of case is considered as a condition, while the concepts in the body of that case are considered decisions. (See parsing example in Table 1(E));
- Call statement: If a decision originates from the sub MLM, then the sub MLM is first parsed in reference to caller MLM through the ID. The executed paths of both caller and called MLMs are concatenated into one path, and the conditions are connected to each other accordingly. (See parsing example in Table 1(F)).

**Table 1:** Control Structure Parsing Examples.

Control Structure Parsing Examples		
A	IF (C = “v1”) THEN	**Condition sentence:** C = “v1”
	D = “d1”	
	Output: “d1 is recommended”	**Decision sentence:** D = “d1”
	END IF	
B	IF (C = “v1”) THEN	For CDSS output “d1 is recommended”:
	D = “d1”	**Condition sentence:** C = “v1”
	Output: “d1 is recommended”	**Decision sentence:** D = “d1”
	ELSE	For CDSS output “d2 is recommended”:
	D = “d2”	**Condition sentence:** C != “v1”
	Output = “d2 is recommended”	Where “!” represents the negation (not).
	END IF	**Decision sentence:** D: d2
C	IF (C = “vl”) THEN	For CDSS output “d1 is recommended”:
	D = “d1”	**Condition sentence:** C = “v1”
	Output: “d1 is recommended”	**Decision sentence:** D = “d1”
	ELSEIF (C in (“v2”, “v3”)) THEN	For CDSS output “d2 is recommended”:
	D = “d2”	**Condition sentence:** C in (“v2”, “v3”)
	Output: “d2 is recommended”	**Decision sentence:** D = “d2”
	ELSEIF (C = “v3”) THEN	For CDSS Output “d3 is recommended”
	D = “d3”	**Condition sentence:** C = “v3”
	Output = “d3 is recommended”	**Decision sentence:** D = “d3”
	ELSE	For CDSS output “d4 is recommended”
	D = “d4”	**Condition sentence:** C != “v3”
	Output = “d4 is recommended”	**Decision sentence:** D = “d4”
	END IF	
D	IF (C1 = “v1”) THEN	**Condition sentence:** C = “v1” AND C2 = “v2”
	IF (C2 != “v2”) THEN	
	D = “d1”	
	Output = “d1 is recommended”	**Decision sentence:** D = “d1”
	END IF	
	END IF	
E	Switch C	For CDSS output “d1” is recommended:
	case v1	**Condition sentence:** C = “v1”
	D = “d1”	**Decision sentence:** D = “d1”
	Output = “d1 is recommended”	For CDSS output “d2 is recommended:
	case v2	**Condition sentence:** C = “v2”
	D = “d2”	**Decision sentence:** D = “d2”
	Output = “d2 is recommended”	
	EndSwitch	
F	IF (C1 = “v1”) THEN	**Condition sentence:** C1 = “v1” AND C2 = “v2”
	Call subMLM1	
	END IF	
	subMLM	
	IF (C2 = “v2”) THEN	**Decision sentence:** D = “d2”
	D = “d2”	
	Output: “d2 is recommended”	
	END IF	

### 4.2. Operator Parsing and Concept Extraction

Operator parsing is the next step after structure parsing. In Arden Syntax, there is a pool of operators. For this work, we only parse the commonly used operators such as “or,” “and,” “=,” “eq,” “is,” “is not equal,” “<>,” “ne,” “is in,” and “in.” The operators “=,” “eq,” and “is” are all parsed as equivalent to “=.” Similarly, “is not equal,” “<>,” and “ne” are parsed as equivalent to the “!=” operator. The binary operators “is in” and “in” are parsed by including OR among the operands. Finally, the logical operators “and,” “or,” and “not” are parsed in the same order in which they occurred. The logical operator “not” has a key role in excluding the undesired elements from the retrieval set. Based on operator parsing, concepts are extracted as operands of the parsed operators for query construction.

### 4.3. Query Construction

Structure parsing involves the use of two lists of concepts, condition concepts and decision concepts, which are interrelated based on the operators. Operator parsing tracks how the concepts are interrelated. In the extraction phase, the concepts are extracted along with location information with respect to operators and structures. Query construction utilizes this tracking to connect the concepts to each other in order to construct the final query. Three types of queries can be made from the final query: condition-based queries, decision-based queries, and combined queries. Condition queries are constructed from the condition concepts, decision queries consist of only decision concepts, and combined queries combine the condition and decision concepts. Each category has its own objective, with advantages and disadvantages. The scope of the proposed work in this paper is limited to combined query construction and evaluation as it involves more information than other two individual types. More formally, the query construction process can be described in Table 2.

**Table 2:** Formal representation of query construction.

*Let* [*Condition*, *Decision*, *Query*] *be set of condition concepts*, *set of decision concepts, and query*
c : *Ρ* Condition (power set of condition concepts)
d : *Ρ* Decision (power set of decision concepts)
q : *Query*
decisionPath : c → d
(decisionPath is function of mapping condtion concepts into decision concepts)
R = *Ρ* decisionPath (Rule R is the set of decisionPath)
KB = *Ρ* R (Knowledge Base KB is the set of rules)
er = *Ρ* R (er represents the set of executed rules)
executedDecisionPath : decisionPath
(*executedDecisionPath represents the executed path in er*)
er ⊂KB executedDecisionPath $:=\{\begin{array}{c} p:decisoinPath;\;\exists r_{1},r_{2}\epsilon \;er\;|\\ (dom\;r_{1}\cup \;r_{2})\mapsto ran\;r_{2}\Rightarrow \\ ran\;r_{1}=\phi \wedge dom\;r_{2}\;\subset \;(dom\;r_{1}\cup \;dom\;r_{2})\cdot \\ dom\;p=(dom\;r_{1}\cup \;(dom\;r_{2})\wedge (ran\;p=ran\;r_{2}) \end{array}\}$ $let\text{ α°}∷=\;\left[NOT\right]\;‹c_{i}\epsilon \;dom\;executedDecisionPath›\;|\;$ $\left\{\begin{array}{c} \left[NOT\right]‹c_{i}\epsilon \;dom\;executedDecisionPath›\;AND|OR\\ \left[NOT\right]‹c_{j}\epsilon \;dom\;executedDecisionPath›\; \end{array}\right\}\;$ let β∷= [NOT] ran executedDecisionPath q= α AND β

### 4.4. Query Execution

**Figure 4:**
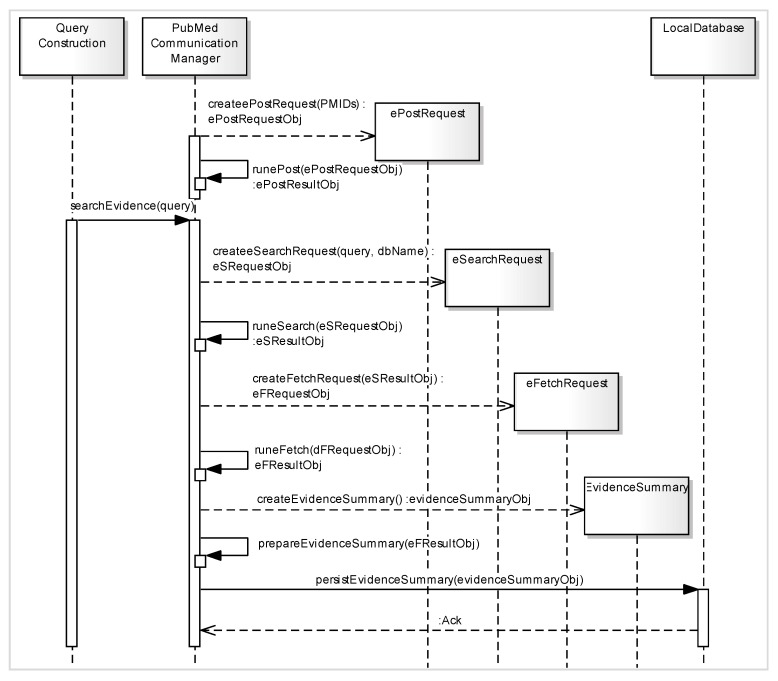
Sequence diagram of eUtils API functions (ePost, eSearch, and eFetch) used as a part of the ***query execution*** function for creation of meta-data associated with evidence.

The constructed query is passed to a URL generator function to generate the URL according to Entrez API for the PubMed search service called Entrez Programming Utilities (eUtils) [31]. The eUtils provide a stable interface to the Entrez query and database system, including 23 databases on a variety of biomedical data. To access these data, a piece of software first posts the eUtils URL to the database in order to retrieve the results. Using eUtils, we build a PubMed URL consisting of a “Base URL” and user query. We also employ the automatic term mapping (ATM) process provided by PubMed [32]. ATM uses translation via MeSH for indexing and searching of the MEDLINE database of journal citations. A neglected term in the query is added to the MeSH term of the original query in order to access the MeSH field of MEDLINE documents. We implement three server functions of eUtils: ePost, eSearch, and eFetch. Using an ePost method, we create our own data set on the PubMed database. The eSearch method searches the relevant documents from the data set. Finally, using eFetch, the meta-information of each retrieved document is extracted, including title, author, journal name, publication year, identifier, and the link to the source document. These functions work in a sequence by using the output of one function as the input for another function. Figure 4 describes the step by step process of different functions involved in the ***query execution*** method in the form of asequence diagram.

## 5. Treatment Recommendation Service Scenario: Results and Evaluation

**Figure 5:**
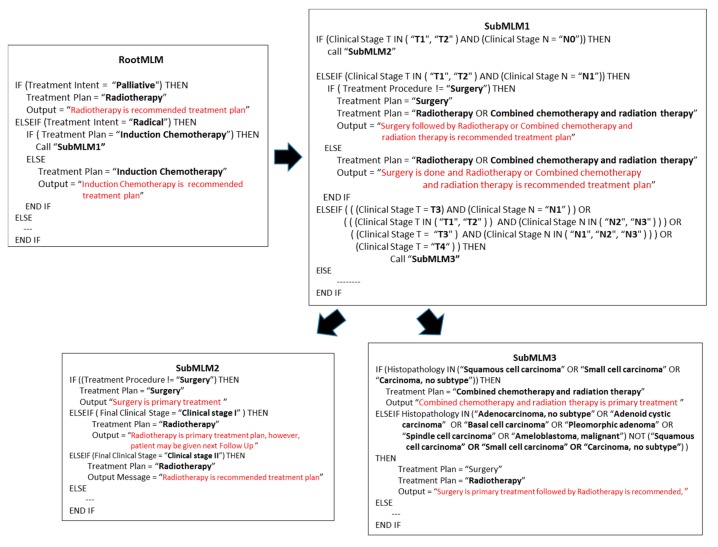
Selected MLMs for the oral cavity site of head and neck cancer embodied with Logic Slots.

The proposed method was tested by applying the knowledge base of Smart CDSS [26,33] to the oral cavity site within the head and neck cancer domain. The knowledge base for the oral cavity site considered for this consists of four MLMs based on a calling mechanism consisting of a sub-MLM approach invoked by the Root MLM. The structure of these MLMs with embodied logic slots is shown in Figure 5 and starts with the Root MLM, followed by “subMLM1,” “subMLM2,” and “subMLM3.” Based on the methodology described in Section 3, the query is constructed from the concepts in the executed logic slot in MLM. Parsing the structure of MLMs according to potential output, 9 outputs were extracted, the structures of which are highlighted in red in Figure 5. Based on these outputs, corresponding queries are generated as shown in Table 3. The queries consists of concepts (highlighted bold in Figure 5) extracted from MLMs and are connected with logical operators according to the logic in the executed path.

**Figure 6:**
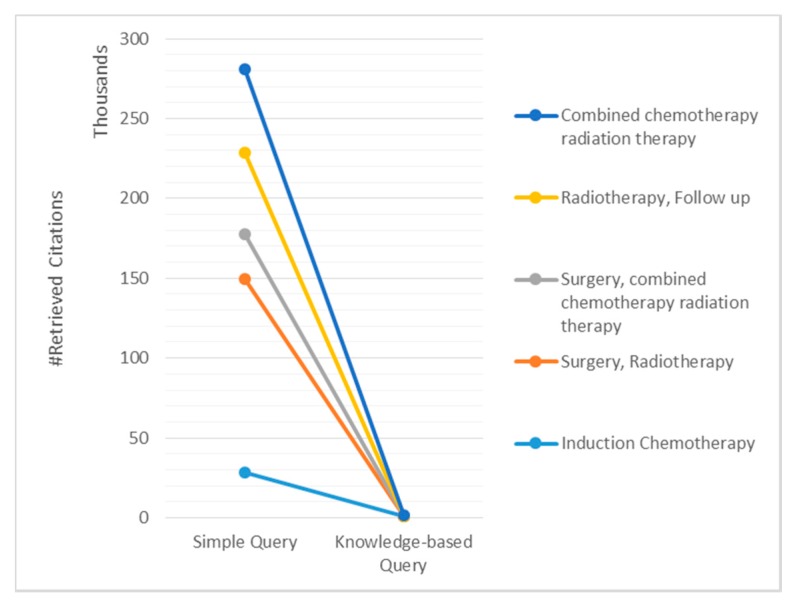
Retrieval set reduction with knowledge-based constructed query compared to simple query.

From the constructed set of queries, we performed experiments on five queries from Table 3 (Q2, Q3, Q4, Q6, and Q8), executed them on a PubMed browser with default setting. We noted a considerable reduction in the resulting citations for knowledge-based constructed queries compared to simple queries, as shown in Figure 6. Simple queries consist of the terms that are used in CDSS output. For instance in Q3 of Table 2, “Surgery” and “Radiotherapy” are the two terms to use in the formation of a simple query. The retrieval set was reduced on average from 56,249 citations to 330 citations. The logical operator “NOT” used in Q9 (Table 3) is derived from SubMLM3 (Figure 5) and has reduced the retrieval set of 1616 documents to just 26 documents by excluding all undesired documents, which indicates its role is crucial to consider while generating the automated query.

**Table 3:** Contents of queries constructed from the executed paths in KB of CDSS outputs.

No	CDSS Output	MLM Reference	Constructed Queries
Q1	Radiotherapy	RootMLM	Palliative AND Radiotherapy
Q2	Induction chemotherapy	RootMLM	Radical and Induction Chemotherapy
Q3	Surgery, radiotherapy	SubMLM1	Radical AND Chemotherapy AND ((T1 OR T2) AND (N1)) AND Surgery AND Radiotherapy
Q4	Surgery, combined chemotherapy radiation therapy	SubMLM1	Radical AND Chemotherapy AND ((T1 OR T2) AND (N1)) AND Combined chemotherapy radiation therapy
Q5	Surgery	SubMLM2	Radical AND Chemotherapy AND ((T1 OR T2) AND (N0)) AND Surgery
Q6	Radiotherapy, follow-up	SubMLM2	Radical AND Chemotherapy AND ((T1 OR T2) AND (N0)) AND Clinical stage I and Radiotherapy and follow up
Q7	Radiotherapy	SubMLM2	Radical AND Chemotherapy AND ((T1 OR T2) AND (N0)) AND Clinical stage II and Radiotherapy
Q8	Combined chemotherapy radiation therapy	SubMLM3	Radical AND Chemotherapy AND (T3 AND N1 ) OR ((T1 OR T2) AND (N2 OR N3 )) OR (T3 AND (N1 OR N2 OR N3)) OR (T4) AND (Squamous cell carcinoma OR Small cell carcinoma OR Carcinoma, no subtype) AND Combined chemotherapy radiation therapy
Q9	Surgery, radiotherapy	SubMLM3	Radical AND Chemotherapy AND (T3 AND N1 ) OR ((T1 OR T2) AND (N2 OR N3 )) OR (T3 AND (N1 OR N2 OR N3) ) OR (T4) AND (Adenocarcinoma, no subtype OR Adenoid cystic carcinoma OR Basal cell carcinoma OR Pleomorphic adenoma OR Spindle cell carcinoma OR Ameloblastoma, malignant) NOT (Squamous cell carcinoma OR Small cell carcinoma OR Carcinoma, no subtype) AND Surgery AND Radiotherapy

**Figure 7:**
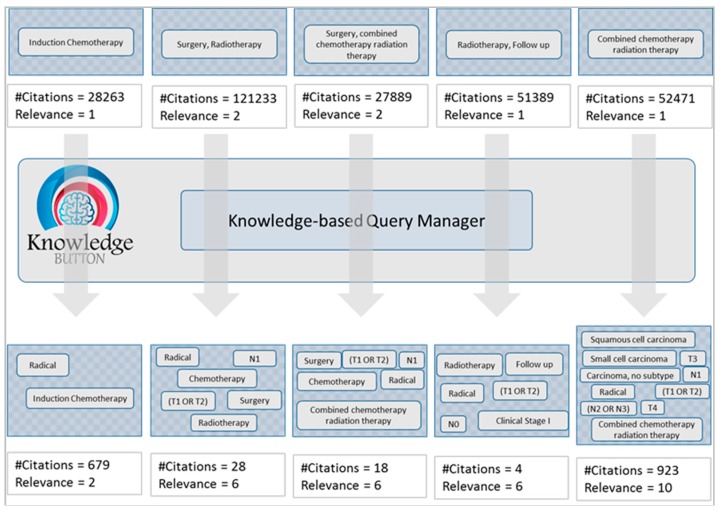
Number of citations is reduced with increased relevance using a knowledge-based query construction mechanism.

The results in Figure 7 shows the terms used in the initial query retrieval set of citations at the upper part. The knowledge-based Query Manager of KnowledgeButton adds more terms extracted from the knowledge rules involved in the CDSS decision. The final knowledge-based queries reduced the retrieval set considerably as shown in the lower part of Figure 7.

The same set of queries used in Figure 7 is counted for relevancy with gold standard documents in order to find the number of documents correctly identified or erroneously omitted by the system. We collected a document set of 131 documents as a gold standard corpus in the head and neck cancer domain with the support of two resident doctors. The gold standard documents are generally related to the head and neck cancer domain without any further classification. We ran the two set queries, simple queries and knowledge-based complex queries, on PubMed search service and matched the results with gold standard documents. The results are evaluated based on the following criteria:

$$recall=\;\frac{\left(relevant\;documents\;retrieved\;\cap^{\text{​}}total\;documents\;retrieved\right)}{total\;relevant\;documents\;(gold\;standard\;documents)}$$

$$Precision=\;\frac{\left(relevant\;documents\;retrieved\;\cap^{\text{​}}total\;documents\;retrieved\right)}{total\;documents\;retrieved}$$

$$F1\;Measure=2\;\times \;\frac{Recall\;\times Precision}{Recall+Precision}$$

As shown in Table 4, precision for knowledge-based queries out-performed the simple queries, but recall for simple queries is shown to be slightly higher than for knowledge-based queries. In terms of overall results, as evident from the F1 score values, the knowledge-based queries showed improved performance and presented the lowest number of documents with a slight compromise on not retrieving some of the relevant documents. On average, the precision of knowledge-based queries is improved by 98.01% at the cost of decreasing recall by only 48.51%. The recall value can be improved by selecting the gold standard set against each query precisely. Also, it can be increased by expansion strategies such as synonyms and variants. We have presented some of these methods in our paper [34].

**Table 4:** Recall, precision, and F1 measure for knowledge-based queries in comparison to simple queries.

Query No.	Query Type	Recall (%)	Precision (%)	F1 Measure (%)
Q2	Simple Query	27.48	0.13	0.38
	Knowledge-based	18.32	3.44	10.33
Q3	Simple Query	41.22	0.04	0.13
	Knowledge-based	18.32	4.94	14.81
Q4	Simple Query	26.72	0.12	0.37
	Knowledge-based	17.56	9.62	28.87
Q6	Simple Query	41.22	0.10	0.31
	Knowledge-based	19.08	4.36	13.07
Q8	Simple Query	42.75	0.11	0.32
	Knowledge-based	19.08	2.67	8.02

**Figure 8:**
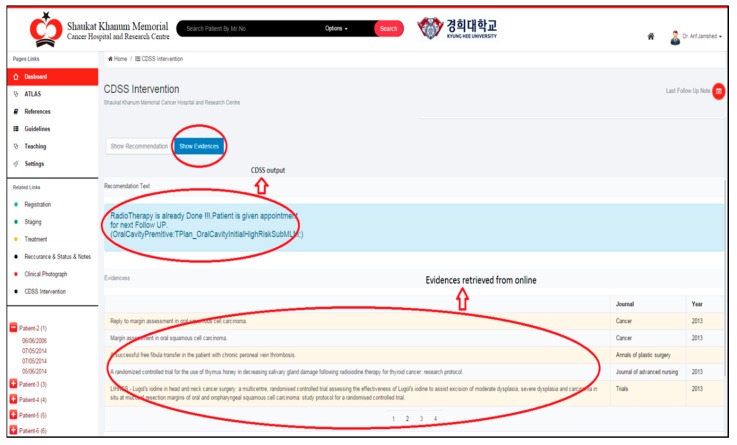
Query result integration with Smart CDSS through KnowledgeButton.

The proposed method was implemented as a part of Smart CDSS integrated with in-house build hospital information system [35] for testing, and the user interface is as described in Figure 8 for users to access the retrieved citations and appraise them as evidence. The documents are then ranked, and the system generates meta-information on ranked documents to store them locally according to knowledge rules. In the future, if the same output from CDSS is generated, the system populates the meta-information from the local repository and retrieves new evidence from the last search. This integrated environment reduces clinical practice time spent unnecessarily on searching in dis-integrated environments.

**Figure 9:**
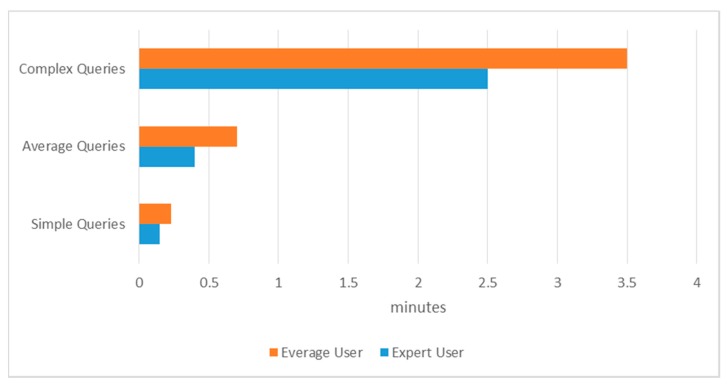
Manual query writing time in minutes for expert and average users.

Automatic query construction saved considerable time compared to manual query construction. An expert clinician can write an average query in around 1 to 2 min excluding the switching time. To measure the time quantitatively, we performed experiments to determine the time needed to manually write three types of queries with simple (consisting of <3 terms), average (consisting of between 4 and 8 terms), and complex (consisting of >8 terms) queries. The experiment was performed with two kinds of users, expert and average. An expert has good domain knowledge and adequate skill in typing, while an average user possesses average domain knowledge and adequate typing skill. We executed the experiment by printing the corresponding queries on paper, opened the PubMed browser to design the query, and recorded the time spent. As shown in Figure 9, the average expert user spent 1.3 min on query construction, while the average user spent 1.5 min. During the experiment, we ignored the mistakes made during writing. The proposed automated query generation has improved performance time by 45.24%.

A short user satisfaction survey was administered to assess the usability of the proposed approach. The survey included three elements: (i) usefulness of integrated approach; (ii) query content; and (iii) relevance. The tasks are evaluated from five clinicians (one consultant, two resident doctors, and two nurses in the oncology domain). The usefulness of an integrated approach was evaluated in comparison to the PubMed browser. Query content was evaluated based on the terms and operators included in the query, and relevance was measured based on the results returned by executing the queries. The impression criterion was rated from very negative to very positive and scored from 1 to 5, respectively, as shown in Table 5.

**Table 5:** User satisfaction based on overall impression for each task with the proposed approach (1= very negative, 5 = very positive).

Task	Expert 1	Expert 2	Expert 3	Expert 4	Expert 5
Usefulness of approach	4	5	3	5	4
Query content	5	4	4	3	4
Relevance of results	4	3	2	4	5

## 6. Evidence Support Services in Ambient Assisted Ubiquitous Environment: A Case Study

To elaborate the utilization of evidence support services in smart environments, we redesigned the workflows in our previous work [26] to support patient medication management in a multi-model sensor home environment for dementia disease patients. In existing work, Smart CDSS was integrated as a recommendation service in a smart home environment where patients are monitored using different sensors.

**Figure 10:**
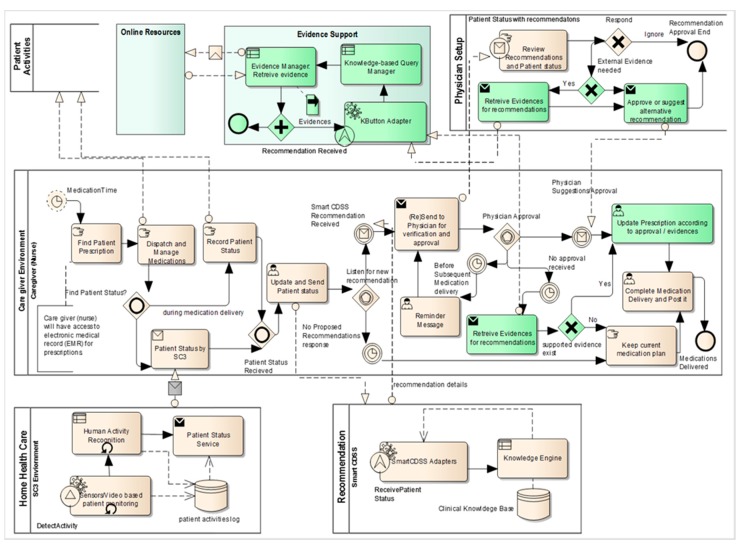
Evidence-supported medication recommendation service workflow represented in Business Process Model and Notation process model where the set of activities is represented as a pool using Enterprise Architect.

As shown in Figure 10, the workflow is categorized into different pools such as home health care, patient activities, care giver environment, recommendation, and physician setup. We enhanced the pool set by adding two additional pools, *i.e.*, evidence support and online resources, in addition to partial changes in existing pools such as physician setup and caregiver environment (changed/enhanced parts are shown in light green color in Figure 10). The detailed descriptions of existing pools are provided in [26]; here we explain only the changed/extended parts with respect to evidence support that involves the process of automatic query construction.

Case 1:

- When the caregiver receives the recommendation generated by Smart CDSS, he/she forwards the information to physicians for approval.
- In normal scenarios, physicians either approve or do not approve the decision made by the system. The caregiver follows the instructions provided by the physician accordingly.
- Sometimes the physician fails to provide any response within a specific time. In such a case, the caregiver checks the criticality of recommended medication and seeks to retrieve evidence that supports the current clinical situation by activating the KButton (KnowledgeButton) Adapter in the evidence support pool.
- In the evidence support pool, the knowledge-based query manager creates a query automatically from the terms/concepts used in the rules involved in generating the recommendation.
- The query is passed to the evidence manger to retrieve relevant evidence from online resources through the use of the PubMed eUtils API.
- The retrieved evidence is presented to the caregiver for to build confidence in deciding on mediation.

Case 2:

- When the recommendation is sent for approval, the physician usually makes a decision based on his or her knowledge and experience; however, sometimes physicians need to verify their knowledge with the work of other experts and researchers.
- In such a case, the physician also activates the KButton (KnowledgeButton) Adapter in the evidence support pool.
- The physician examines the evidence (if any) and then proceeds with approval of the recommendation or may suggest alternatives.

## 7. Discussion

This work is performed in the context of CDSS to provide clinicians with facility of retrieving relevant evidences from external resources. We particularly focused on automatic construction of queries using the complex structure of MLM in Arden Syntax. Clinicians who evaluated the prototype were enthusiastic, but they highlighted an important aspect of such a system regarding consistency in retrieval efficiency in terms of relevancy. The approach proposed in this paper is different from existing approaches [13,23,36,37] with regard to the source from which the query is constructed. Some of the existing systems utilize documents as a training set for query construction, and others use patient records. For instance, Infobuttons operates on pre-specified query topics associated with important terms in the electronic medical record. In our approach, the query is automatically created from the terms used in knowledge rules that participate in the CDSS decision. We utilized the standard but complex structure of MLM to automatically construct queries. This work improves the effectiveness of clinical knowledge-based systems by coupling with external resources.

The proposed work introduced a novel approach to knowledge-based complex query construction using the diagnostic knowledge in CDSS to retrieve research-based evidence from online resources. Diagnostic knowledge alone is not sufficient to satisfy CDSS users, who expect ample details to support a decision. For example, a clinical-stage attribute used in a condition part of the rule together with other condition attributes produces a decision, but details such as tumors and nodes may be missing. Our hypothesis is that fine-grained details of the missing attributes might be found in credible external resources. If such resources are used as support for the rules used in diagnostic knowledge, practitioners will be able to retrieve detailed, readily available knowledge.

The proposed work has a few limitations, e.g., the “structure parsing” method is unable to parse all the control structures, such as the “loop control structure”. It also does not parse all the operators of HL7 Arden Syntax such as “string operators” or “aggregate operators” for query construction. We included the most important and usable logical operators, but as an extension of this work it would be necessary to investigate other operators as well as control structures of Arden Syntax. The overall impression of users was recorded on initial implementation and needs to be re-evaluated after using the system in multiple sessions to satisfy different clinical questions.

## 8. Conclusions

Automatic construction of knowledge-based complex queries is an important task needed to reduce clinical time in the context of evidence-based practice. The proposed work describes the methodology of automatic query construction using the control structure and operator parsing of Arden Syntax medical logic modules. Adding context from knowledge rules maximizes the relevance while reducing the number of retrieved citations as inferred from the experiments. Our results show higher precision and F1 score for all tested queries. On average, the F1 measure of knowledge-based queries is improved by 97.01%. Similarly, the proposed work of automated query generation has improved time performance by 45.2%. The proposed work bridges the gap in clinical practice by integrating the diagnostic knowledge of the clinical decision support system with research evidence through automation of the construction of knowledge-based queries.

Our plan for future study is to improve retrieval performance by including semantic similarity methods and concrete appraisal of retrieved documents. Also, we will focus on enhancing the parsing of Arden Syntax control structures and operators for query generation.
